# Sex differences in cardiometabolic risk factors and in response to lifestyle intervention in prepubertal and pubertal subjects with obesity

**DOI:** 10.3389/fped.2024.1304451

**Published:** 2024-02-12

**Authors:** Luisa Gilardini, Marina Croci, Luca Cavaggioni, Lucia Pasqualinotto, Simona Bertoli

**Affiliations:** ^1^ Obesity Unit—Laboratory of Nutrition and Obesity Research, Department of Endocrine and Metabolic Diseases, IRCCS Istituto Auxologico Italiano, Milan, Italy; ^2^ International Center for the Assessment of Nutritional Status (ICANS), Department of Food, Environmental and Nutritional Sciences (DeFENS), University of Milan, Milan, Italy

**Keywords:** childhood obesity, gender, lifestyle intervention, cardiometabolic risk factors, pubertal stage

## Abstract

**Objectives:**

Childhood obesity is a growing health problem and requires a tailored treatment. This study explored the sex differences in cardiovascular risk factors in children/adolescents with obesity and in response to a weight loss intervention.

**Methods:**

Five hundred and thirty-three children/adolescents with obesity and their parents underwent to a 3-months lifestyle intervention program. Tanner criteria were used to assess the pubertal stage. Before and after 3 months, anthropometric measurements, blood pressure (BP), and biochemical measurements were assessed.

**Results:**

Four hundred and forty five participants completed the treatment (age 12.4 ± 2.7 years, males 45.8%, prepubertal 29.2%, BMI *z* score 2.3 ± 0.2). In comparison to boys, prepubertal girls had higher values of BMI *z* score (2.4 ± 02 vs. 2.3 ± 0.2, *p* < 0.05), waist circumference *z* score (2.2 ± 0.3 vs. 2.0 ± 0.3, *p* < 0.05), HOMA-IR [2.9 (2.1–4.9) vs. 2.3(1.5–3.6), *p* < 0.01], prevalence of hypertransaminasemia (41.3% vs. 17.7%, *p* < 0.05) and lower levels of HDL cholesterol (46.2 ± 9.8 vs. 51.2 ± 10.5 mg/dl, *p* < 0.05). In the pubertal stage, boys had worse cardiometabolic risk profile than girls, including unfavourable measure of systolic BP (*z* score: 0.6 ± 1.0 vs. 0.3 ± 1.0, *p* < 0.01), fasting glucose (87.2 ± 6.1 vs. 84.8 ± 7.7 mg/dl, *p* < 0.01), ALT (26.9 ± 21.5 vs. 20.2 ± 10.6 U/L, *p* < 0.001) and uric acid (6.1 ± 1.9 vs. 5.0 ± 1.0 mg/dl, *p* < 0.001). After the lifestyle intervention, changes in BMI *z* score (*p* < 0.05) were higher in pubertal boys than pubertal girls. The systolic blood pressure decrease was greater in pubertal boys than in their female counterpart (Δ systolic BP: −7.2 mmHg in boys vs. −3.6 mmHg in girls, *p* < 0.05; Δ systolic BP *z* score: −0.6 in boys vs. −0.3 in girls, *p* < 0.05). LDL cholesterol showed an improvement only in boys, and ALT in the whole group.

**Conclusion:**

Our study showed that a short-term lifestyle intervention is more effective in reducing BMI *z* score and cardiovascular risk factors in pubertal boys than in their female counterparts. Further investigation is needed to deepen this gender difference, especially to develop a tailor-made intervention.

## 1 Introduction

High-income Western countries have seen a significant increase in the prevalence of overweight and obesity in children over the past 40 years, with obesity affecting one-sixth of children and adolescents aged 5–19 years in 2015 (1). In high- and upper middle-income countries, the obesity prevalence seems to be greater in boys than in girls (2), but the possible causes and consequences of this difference are not fully comprehended. Childhood obesity is a significant public health concern, as fat accumulation at a younger age increases the risk of premature cardio-metabolic complications, including type 2 diabetes mellitus, dyslipidemia, and hypertension, which lead to a significant risk of developing cardiovascular disease in adulthood (3). Given the rather alarming data, several European countries have adopted preventive programs to face the problem at a national level, monitoring overweight and obesity and their related risk factors and promoting healthy habits (4).

In adult age, several obesity-related comorbidities, including type 2 diabetes and hypertension, exhibit sex-specific pathways. The prevalence of diabetes in men is higher than in women, particularly in middle-aged adults, and men develop diabetes at a lower BMI than women (5). Impaired fasting glucose is more prevalent in men while impaired glucose tolerance is more common in women (6). Moreover, the impact of obesity on hypertension is different in the two sexes as a 3-unit increase in BMI for obese women resulted in a higher increase in systolic blood pressure compared with men (5).

The role that sex/gender may play in childhood obesity and related cardiovascular risk factors has not been completely studied. In addition to gender, we must consider that body fat distribution, insulin resistance, blood pressure, and lipids are all affected by puberty and normal growth (7).

Weight loss is the main way to reduce the risks associated with obesity and the guidelines indicate that family-based lifestyle interventions, such as dietary modifications and increased physical activity, are the primary approach to weight management in children (8). Weight loss programs have varying results and the sex-based response to lifestyle changes has not been fully explored in childhood obesity.

The purpose of this study is to determine (1) the effect of gender in cardiovascular risk factors in children/adolescents with obesity in different pubertal stage and (2) whether the effectiveness of a weight loss intervention in children/adolescents with obesity differs between boys and girls.

## 2 Methods

A 3-month lifestyle intervention program, dedicated to children/adolescents with obesity and their families, has been conducted for many years at the Istituto Auxologico Italiano in Milan. The treatment is provided by the national healthcare system and the entire program was approved by the local health authorities. We have implemented a database with the data of the subjects who consecutively attended this program between 2008 and 2019. The program has not undergone substantial changes in the period studied. Part of this database was used, in collaboration with other study groups, for two previous publications in which was investigated the association of visceral adiposity index and metabolic syndrome and the relation of metabolically healthy and unhealthy obesity with cardiovascular risk factors in children (9, 10). For the aim of this study, we considered subjects 6–18 years aged and without secondary obesity, binge eating disorders and on any drug therapy.

The International Obesity Task Force definition of childhood obesity was used to assess obesity and the BMI *z*-score was calculated using Cole's least mean square method (11).

### 2.1 Clinical assessment

Family history of obesity, birthweight, and duration of overweight were assessed by questionnaires filled in by parents. Before and after the 3-month lifestyle intervention, anthropometric measurements, blood pressure (BP), and biochemical measurements were assessed.

Body weight was recorded to the nearest 0.1 kg using a standard beam balance scale, with the subjects wearing light indoor clothing and no shoes. Height was recorded to the nearest 0.5 cm using a standard wall-mounted height board. Waist circumference was measured in standing position at the midpoint between the last rib and the iliac crest with a non-stretch tape to the nearest 0.5 cm. The waist circumference *z* score was based on tables using the National Health and Nutrition Survey III (12). Pubertal development was assessed by physical examination according to the criteria of Tanner (13). Subjects with Tanner stage I were defined as prepubertal, while those with stage II–V were defined as pubertal. BP was measured using an aneroid sphygmomanometer with the appropriate cuff for the child's upper arm size, after at least 5 min of rest. Korotkoff sounds-based measurement has been used to assess systolic BP (K1) and diastolic BP (K5). BP measures were taken 3 times (at 3–5 min intervals) and the average of the two last SBP and DBP measurements was calculated. Systolic and diastolic BP percentiles were calculated according to the normograms of the National High Blood Pressure Education Program (NHBPEP) Working Group on High Blood Pressure in Children and Adolescents (14).

A blood sample was drawn for the measurement of LDL and HDL cholesterol, triglycerides, uric acid and alanine aminotransferase (ALT), and glucose and insulin during an oral glucose tolerance test. A central laboratory at the Istituto Auxologico Italiano was used for all determinations. Biochemical measurement methods did not change between 2008 and 2019. Insulin resistance was estimated using the homeostasis model assessment for insulin resistance (15). A subject was insulin resistant if HOMA-IR was >75th percentile of age- and sex-adjusted reference levels. The percentiles were derived from a large group of Italian overweight/obese children (16).

High triglycerides were defined by plasma levels ≥100 mg/dl for children aged 7–9 years and ≥130 mg/dl for adolescents aged 10–18 years. High LDL cholesterol was defined by plasma levels ≥130 mg/dl and low HDL cholesterol was defined by plasma levels <40 mg/dl (17).

High alanine aminotransferase was defined by ALT serum concentrations >25 U/L in boys and >22 U/L in girls (18). The impaired fasting glucose (IFG), impaired glucose tolerance (IGT), and type 2 diabetes were defined according to American Diabetes Association criteria (19).

### 2.2 Lifestyle intervention

The intervention program was multidisciplinary and consisted of one session per week, with nutrition education, physical exercise, and behavioral therapy. Each session lasted about four hours and involved the child and family. At the first visit the dietician, after having collected the dietary history, provided a diet ranging from 1,600 to 2,000 kcal/die, rich in vegetables and low in salt and simple sugars. The proportions of carbohydrate, protein, and fat in the diet were 55%, 15% and 30%, respectively. At each visit, the child and parents met with the dietitian who verified adherence to the diet, provided strategies for managing critical issues, and encouraged the change of unhealthy eating habits. A self-monitor diary was used as a tool for education and reinforcement. In this diary food consumption, daily physical activity, and emotional reactions were reported. The nutritional intervention included seven educational group sessions for children and one for parents. These meetings were structured around an interactive teaching/learning approach and focused on various topics, such as healthy eating principles, the Mediterranean food pyramid, and the strategies for managing meals out and social events. At each session, the children attended an hour of physical activity in the gym, under the supervision of an exercise physiologist, who monitored that physical activity was performed correctly. The activity consisted of moderate-intensity aerobic physical activity and muscle-strengthening activities. In detail, the session was formed by continuous cardiorespiratory fitness on a cycle ergometer (20 min) and a whole-body, circuit-training muscle-strengthening exercises (20 min) with the aim to improve fundamental movement patterns (e.g., squat, hinge, lunge, push, pull, carry, rotation), postural control and joint stability. Additionally, a 10-min warm-up and cool-down periods, composed by mobility, motor coordination, and static streching, were also included in each session. The psychological intervention consisted of at least two interviews preferably with the family, to assess the presence of an eating disorder or potential risk factors for it. Additional counseling with the entire family may be conducted based on the need. The intervention included four psycho-educational groups with the children and three with the family. The groups examined dysfunctional nutrition behaviors and developed strategies to limit and eliminate them.

### 2.3 Statistical analysis

All results are expressed as mean (standard deviation), percentage, or median (interquartile range), if variables were not normally distributed (HOMA-IR, triglycerides). These latter were log-transformed for the analysis.

Analysis of variance was carried out to compare differences among groups in baseline values. The group frequencies were compared using a chi-square test.

Paired *t*-test was used to compare variables before and after intervention. The changes induced by lifestyle intervention were calculated using the following ratio: value at month 3—baseline value. ANCOVA was used to test the sex difference in these changes, using baseline value and age as covariates. To evaluate if the BMI *z* score changes correlated with the improvement of cardiovascular risk factors, Pearson correlation analysis was used. The significance level for all tests was set at *p* < 0.05. All analyses were performed using SPSS version 28.0 (Statistical Package for Social Science Inc., Chicago, Ill, USA).

## 3 Results

Five hundred and thirty-three subjects were admitted to the lifestyle modification program and 445 participants completed the treatment (age years). There were not significant differences in age (12.4 ± 2.7 vs. 12.1 ± 3.1 years, ns), sex (males 45.8 vs. 42.0%), pubertal stage (prepubertal 29.2 vs. 32.6%, ns) and degree of obesity (BMI *z* score 2.3 ± 0.2 vs. 2.3 ± 0.3, ns) between subjects who completed or did not complete the intervention.

The subsequent analyses were carried out only in the 445 subjects who concluded the program.

**Figure 1:**
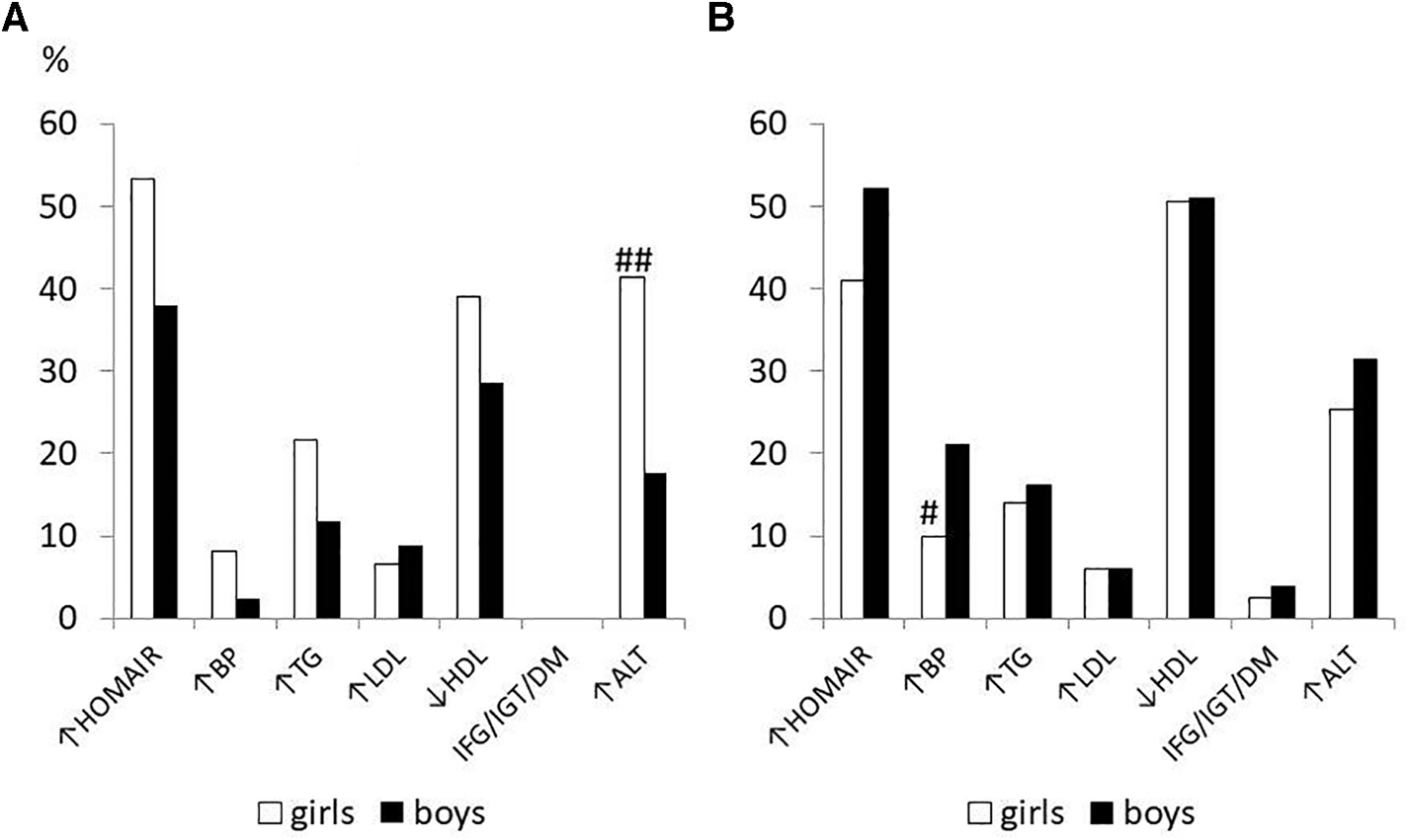
Prevalence of cardiometabolic risk factors at baseline in 455 children/adolescents with obesity in prepubertal (**A**) and pubertal stage (**B**) divided by sex # *p* < 0.05, ##*p* < 0.01 vs. boys.

Figure 1 described the baseline gender differences in the prevalence of cardiovascular risk factors in subjects, divided by pubertal stage. In the prepubertal stage, girls had a significantly greater prevalence of high transaminase than boys, instead in the pubertal stage boys were significantly more hypertensive than their female counterparts. The abnormalities of glucose metabolism were present only in the pubertal stage and were distributed in this way: a 14-year-old girl had unknown diabetes (subsequent investigations have diagnosed type 1 diabetes), 4 subjects (2 girls and 2 boys) had IGT and 5 had IFG (2 girls and 3 boys).

Tables 1, 2 show the anthropometric variables and cardiovascular risk factors in prepubertal (Tanner stage I) and pubertal (Tanner II–V) subjects respectively, divided by sex at baseline and at the end of the intervention.

**Table 1:** Anthropometric and clinical variables before and after the lifestyle modification program in prepubertal subjects (tanner stage I) divided by sex.

	Girls		Boys	
	Baseline	3 months	Baseline	3 months
Age, years	8.6 ± 1.4^°°^		9.8 ± 1.3	
FH for obesity, %	73.9		77.2	
FH for diabetes, %	61.7		62.0	
FH for CVD, %	44.7		40.5	
BMI, kg/m^2^	27.5 ± 3.2	26.5 ± 3.2*	27.8 ± 3.1	26.8 ± 3.2*
BMI *z* score	2.39 ± 0.2^°^	2.28 ± 0.2*	2.30 ± 0.2	2.20 ± 0.2*
Waist circ., cm	88.4 ± 9.7	85.7 ± 8.9*	91.5 ± 10.2	90.3 ± 9.6*
WC *z* score	2.2 ± 0.3^°^	2.1 ± 0.3*	2.0 ± 0.6	1.9 ± 0.3*
Systolic BP, mmHg	97.3 ± 10.4^°°°^	97.0 ± 8.0	104.1 ± 11.6	101.1 ± 8.6**
Diastolic BP, mmHg	67.0 ± 7.3^°^	67.9 ± 5.5	69.8 ± 7.0	68.7 ± 5.6
Systolic BP (*z* score)	−0.5 ± 0.9	−0.5 ± 0.6	−0.1 ± 0.9	−0.3 ± 0.7**
Diastolic BP (*z* score)	0.6 ± 0.6	0.6 ± 0.5	0.7 ± 0.6	0.6 ± 0.5
Fasting glucose, mg/dl	84.5 ± 5.8	83.4 ± 4.3	83.6 ± 5.0	82.3 ± 5.6
HOMA-IR	2.9 (2.1–4.9)^°°^	2.7 (2.2–4.7)	2.3 (1.5–3.6)	2.3 (1.6–3.4)
LDL cholesterol, mg/dl	94.5 ± 22.9	94.3 ± 19.6	95.3 ± 23.8	91.7 ± 22.3**
HDL cholesterol, mg/dl	46.2 ± 9.8^°^	45.9 ± 9.6	51.2 ± 10.5	50.4 ± 11.0
Tryglicerides, mg/dl	79.0 (61.0–111.5)	74.0 (53.0–99.0)	66.0 (49.0–90.5)	67.5 (48.5–101.0)
Uric acid, mg/dl	4.4 ± 0.9	4.2 ± 0.9***	4.5 ± 0.9	4.4 ± 0.9
ALT, U/L	24.4 ± 13.1	19.4 ± 10.6*	21.4 ± 12.1	19.7 ± 9.1***

FH, family history; WC, waist circumference; BP, blood pressure.

* *p* < 0.001.

** *p* < 0.01.

*** *p* < 0.05 vs. baseline.

^°^ *p* < 0.05.

^°°^ *p* < 0.01.

^°°°^ *p* < 0.0001 vs. boys at baseline.

**Table 2:** Anthropometric and clinical variables before and after the lifestyle modification program in pubertal (Tanner stage II–V) subjects divided by sex.

	Girls		Boys	
	Baseline	3 months	Baseline	3 months
Age, years	13.6 ± 2.2		13.7 ± 2.2	
FH for obesity, %	75.4		76.0	
FH for diabetes, %	64.1		60.3	
FH for CVD, %	37.8		40.8	
BMI, kg/m^2^	33.6 ± 3.6	32.6 ± 3.7*	33.1 ± 4.7	31.9 ± 4.6*
BMI *z* score	2.26 ± 0.2^°°^	2.18 ± 0.2*	2.33 ± 0.2	2.23 ± 0.2*
Waist circumference, cm	104.2 ± 10.4^°°^	101.2 ± 10.7*	109.3 ± 12.1	105.6 ± 12.3*
WC *z* score	2.0 ± 0.3	1.9 ± 0.3*	2.0 ± 0.2	1.9 ± 0.2*
Systolic BP, mmHg	112.3 ± 11.7^°°^	108.7 ± 9.7*	119.3 ± 12.7	111.6 ± 109*
Diastolic BP, mmHg	73.0 ± 6.7^°^	71.2 ± 6.4**	75.2 ± 7.3	72.7 ± 6.1***
Systolic BP (*z* score)	0.3 ± 1.0^°^	−0.0 ± 0.9*	0.6 ± 1.0	−0.1 ± 0.9*
Diastolic BP (*z* score)	0.7 ± 0.6	0.6 ± 0.6**	0.9 ± 0.6	0.7 ± 0.5***
Fasting glucose, mg/dl	84.8 ± 7.7^°^	84.6 ± 6.6	87.2 ± 6.1	86.9 ± 5.7
HOMA-IR	3.3 (2.5–4.8)	3.6 (2.5–5.0)	3.6 (2.5–4.7)	3.6 (2.5–5.0)
LDL cholesterol, mg/dl	95.2 ± 23.4	92.9 ± 22.9	95.8 ± 24.4	92.7 ± 21.7***
HDL cholesterol, mg/dl	45.5 ± 8.8	44.8 ± 8.6	45.7 ± 9.9	45.2 ± 9.8**
Tryglicerides, mg/dl	78.0 (60.0–101.2)	80.0 (64.0–110.0)	79.0 (56.2–106.0)	84.0 (59.0–111.5)
Uric acid, mg/dl	5.0 ± 1.0^°^	5.0 ± 3.6	6.1 ± 1.9	5.9 ± 1.3***
ALT, U/L	20.2 ± 10.6^°^	18.6 ± 9.5***	26.9 ± 21.5	24.4 ± 19.2**

FH, family history; WC, waist circumference; BP, blood pressure.

* *p* < 0.001.

** *p* < 0.01.

*** *p* < 0.05 vs. baseline.

^°^ *p* < 0.01.

^°°^ *p* < 0.001 vs. boys at baseline.

In the prepubertal group, girls had higher BMI and waist circumference *z* scores and HOMA-IR than boys and lower systolic blood pressure and HDL cholesterol. The differences in HOMA-IR and HDL cholesterol remained also after adjustment for age and BMI and waist circumference *z* scores (HOMA-IR: 3.1 (2.3–5.1) vs. 2.5 (1.7–3.8), *p* < 0.001; HDL cholesterol: 45.4 ± 8.7 vs. 51.8 ± 10.9 mg/dl, *p* < 0.01).

In the pubertal group, girls had lower BMI *z* score, systolic blood pressure, fasting glucose, uric acid, and transaminase than boys. All differences remained also after adjustment for BMI *z* score (fasting glucose: 84.9 ± 7.8 vs. 87.0 ± 5.9, *p* < 0.05; uric acid: 5.1 ± 1.0 vs. 6.0 ± 1.9, *p* < 0.0001; ALT: 20.6 ± 10.5 vs. 26.3 ± 21.1, *p* < 0.01), except for systolic blood pressure.

**Figure 2:**
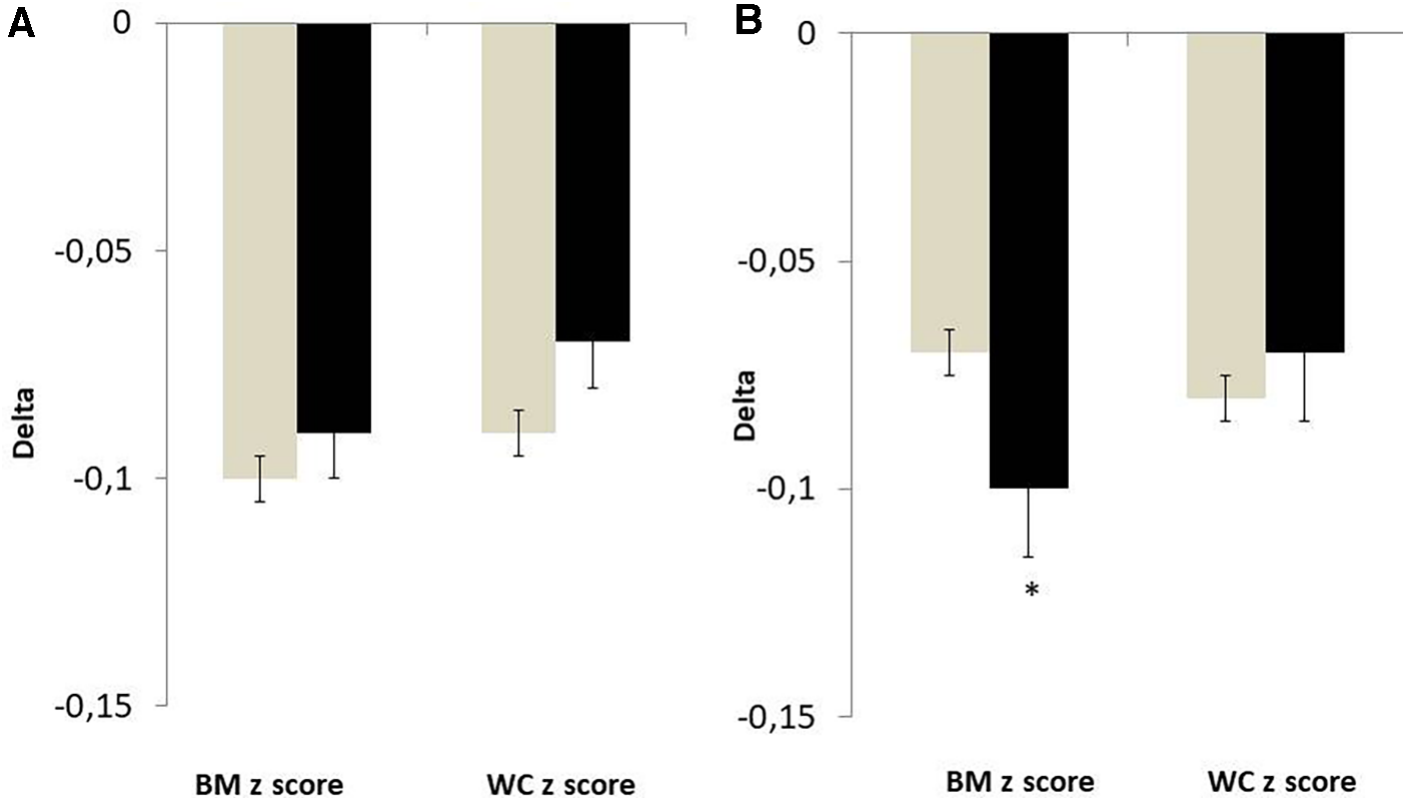
Changes in anthropometric variables in girls (grey bar) and in boys (black bar) divided by pubertal stage. Values were adjusted for baseline value and age. (**A**) Tanner stage I. (**B**) Tanner stage II–V. *p < 0.05 vs. girls.

After 3 months of lifestyle modification intervention, the mean relative BMI *z* score decrease was −3.9 ± 4.4% (*p* < 0.001) in the whole group. The decrease in BMI *z* score and waist circumference *z* score were significant vs. baseline in prepubertal and pubertal boys and girls (*p* < 0.001 for all). After adjustment for age and baseline value, changes in BMI *z* score were higher in pubertal boys than pubertal girls (Figure 2). There were no gender differences in anthropometric changes in the prepubertal subjects.

Considering prepubertal subjects, girls significantly showed a reduction in uric acid and ALT, while boys a reduction in systolic blood pressure, LDL cholesterol, and ALT (Table 1).

In pubertal subjects, the systolic and diastolic blood pressure and ALT levels have reduced in the two sexes. The levels of LDL cholesterol and uric acid were significantly decreased only in boys (Table 2).

The systolic blood pressure decrease was greater in pubertal boys than in their female counterpart (Δ systolic BP: −7.2 mmHg in boys vs. −3.6 mmHg in girls, *p* < 0.05; Δ systolic BP *z* score: −0.6 in boys vs. −0.3 in girls, *p* < 0.05). The delta of other cardiovascular risk factors (value at 3 months—baseline value) did not show any significant gender differences.

The improvement in blood pressure, ALT, uric acid, and LDL cholesterol did not correlate with the change in BMI *z* score (data not shown).

## 4 Discussion

In our group of children/adolescents with obesity, the most frequent cardiovascular risk factors were insulin resistance (44.8%) and dyslipidaemia (53.1%), while abnormalities of glucose metabolism were rare, with a prevalence of 2.2%. Elevated serum alanine transaminase (ALT) and hypertension were present in 27.8% and 12.6% of the subjects, respectively. The prevalence of insulin resistance was similar to that described in a group of US subjects with obesity (20). Like in this population, in our cohort, HOMA-IR was greater in girls than in boys in the prepubertal stage. In the pubertal stage, HOMA-IR and insulin resistance were higher in boys than in girls, although in no significant way. These findings confirmed a previous longitudinal study by Moran et al. (21) in which insulin resistance, measured by euglycemic insulin clamp, was greater in females than males at age 11 years and lower by late adolescence. Despite the high percentage of insulin resistance in our population, the frequency of abnormalities of glucose metabolism was very low and concerned only the pubertal group. This result is difficult to explain especially because the frequency of glucose metabolism alteration is lower than that found in Italian cohorts of children with the same degree of obesity (22–24). In our study, boys had higher blood glucose than girls in the pubertal stage, confirming the previous data in the literature in both children and adults (20, 21, 6).

In the prepubertal stage, girls seemed to have worse cardiometabolic profile than boys, including an unfavorable measure of BMI *z* score, waist circumference *z* score, insulin resistance, low HDL cholesterol, and high ALT. The opposite occurred in the pubertal stage, where boys showed higher levels of blood pressure, fasting glucose, ALT, and uric acid than girls.

Lifestyle intervention is an efficacious treatment strategy for children with obesity. In our study, significant improvements in BMI *z* score and waist circumference were obtained independently by sex and pubertal stage. Pubertal boys had a greater decrease in BMI *z* score than their female counterparts. A multitude of studies reported a significant effect of lifestyle intervention in children and adolescents with obesity on at least one measure of adiposity (24–26) but few studies focused on the role of sex on intervention outcomes with conflicting results. A study on the effect of a 12-week lifestyle intervention in Latino adolescents with obesity, had results like ours showing that males compared to females had greater reduction in fat mass and gained more fat-free mass (27). A multidisciplinary inpatient program carried out in Switzerland in a small group of adolescents with severe obesity, showed that boys lowered their BMI by a greater extent than girls (28). A recent review analyzed the effect of gender on response to lifestyle intervention among children and adolescent and concluded that boys and girls did not demonstrate a differential response to the treatment for weight, BMI *z* score or waist circumference (29). Most of the study included in this review was not designed to detect sex as a primary determinant of outcomes.

To gain a better understanding of the possible reasons for the different responses to treatment between male and female adolescents, more research is required. For example, it is well known in the literature that there is a gender difference in the practice of physical activity among young people: girls are less physically active than boys. There may also be a gender difference in adherence to the physical activity program, as has been described in two studies on youths with obesity. In an Italian study, a camp experience in children with obesity produced different changes in physical activity levels by sex; whereas male children improved physical activity levels during the camp compared with their levels before camping, females did not change their habits in terms of physical behaviors (30). An intervention study in which obese children wore accelerometers every day for 8 weeks, that compared with girls, boys showed greater increases in overall daily physical activity (31).

After 3 months of lifestyle intervention cardiometabolic risk markers improved. Blood pressure decreased in both sexes, but with some differences. The pubertal boys had the greatest prevalence of hypertension and showed the most effective reduction in blood pressure after lifestyle changes. This finding is particularly important since hypertension in children may perpetuate in adulthood, and Rosner et al. showed that elevated BP at 15 years old is a risk factor for hypertension at 35 y more in males than in females (32).

In our study group, the prevalence of elevated LDL cholesterol levels was low and no different regarding gender and puberty. This agrees with a study in a Spanish cohort of children and adolescents with obesity, that showed that LDL cholesterol did not differ according to sex and a prevalence of elevated LDL similar to ours (33). Only pubertal boys showed an LDL cholesterol decrease at the end of rehabilitation. It is well known that atherosclerosis originates in childhood and LDL cholesterol is the most atherogenic lipoprotein. The early detection and treatment of hypercholesterolemia is crucial to prevent atherosclerosis development (34), especially in males. Indeed, they have a higher incidence of myocardial infarction than females, with males accounting for approximately 70% of infarctions and having a myocardial infarction 7–10 years earlier than females (35).

The increased ALT levels are a surrogate marker for NAFLD, which has now become the most frequent cause of chronic liver disease, both in children and adults, and predicts later development of diabetes and metabolic syndrome in adults (36). Lifestyle changes with a healthier diet, gradual weight loss, and increased physical activity seem to be the first-line treatment for this disease at present and several studies demonstrated the beneficial effect of weight loss on fatty liver disease (37). In a cohort of 117 Danish children with obesity, a 10-week “weight loss camp” induced a significant reduction in transaminase levels, without gender difference (38). The improvement of the transaminase levels after the intervention is particularly important in our cohort because, the increased ALT level was present also at an early age, especially in females.

The improvements in cardiometabolic variables were not related to BMI *z* score changes, demonstrating that the mechanisms at work are complex and need more research. Indeed, the positive effect of the intervention on cardiometabolic risk factors may be attributable to aspects of lifestyle changes that are independent of weight loss, such as the reduction in saturated fat and salt intake or increased physical activity.

The main limitation of the current study is the short duration of the treatment and the lack of follow-up. At the end of the program, subjects were given an appointment for the regular three-month follow-up visits. Our future project is to collect follow-up data to explore whether the benefits of the intervention are maintained in the long term. On the other hand, the relatively short 3-month duration could equally be viewed as a strength since the improvements in cardiovascular risk factors were seen relatively quickly. Secondly, we did not explore the adherence to diet and physical activity. In this regard, in future research, we could use technological tools, that have been demonstrated essential in the management of pediatric chronic diseases during the recent COVID-19 pandemic (39). An accelerometer or an electronic diary could be useful to record both physical activity and food and drink intake. This investigation would allow us to better explain the gender differences in the effectiveness of the intervention since usually males tend to adhere more to the physical activity program and females to have a healthier diet (40). Finally, we are aware that the study population cannot be considered representative of Italian children/adolescents and further investigations are recommended, involving larger and multi-center populations.

In conclusion, our study showed that short-term lifestyle intervention is more effective in reducing BMI *z* score and cardiovascular risk factors in pubertal boys than in their female counterparts. This finding encourages us to deepen the reason for this gender difference and to increase efforts to promote weight loss in females. Moreover, we are satisfied that better results were obtained in males with obesity, since a higher mortality risk for all causes of death, especially atherosclerotic cerebrovascular disease, and colorectal cancer, was demonstrated in males but not in females who were overweight during high school years (41).
